# Strategic larval decision-making in a bivoltine butterfly

**DOI:** 10.1007/s00442-011-2238-z

**Published:** 2012-01-26

**Authors:** Magne Friberg, Josefin Dahlerus, Christer Wiklund

**Affiliations:** 1 Department of Zoology, Stockholm University, 10691 Stockholm, Sweden; 2 Department of Ecology and Evolutionary Biology, Earth and Marine Sciences Building, University of California, Santa Cruz, CA 95064 USA

**Keywords:** Diapause/direct development, Phenotypic plasticity, Life history, Developmental switch, Developmental constraints

## Abstract

In temperate areas, insect larvae must decide between entering winter diapause or developing directly and reproducing in the same season. Long daylength and high temperature promote direct development, which is generally associated with a higher growth rate. In this work, we investigated whether the larval pathway decision precedes the adjustment of growth rate (state-independent), or whether the pathway decision is conditional on the individual’s growth rate (state-dependent), in the butterfly *Pieris napi*. This species typically makes the pathway decision in the penultimate instar. We measured growth rate throughout larval development under two daylengths: slightly shorter and slightly longer than the critical daylength. Results indicate that the pathway decision can be both state-independent and state-dependent; under the shorter daylength condition, most larvae entered diapause, and direct development was chosen exclusively by a small subset of larvae showing the highest growth rates already in the early instars; under the longer daylength condition, most larvae developed directly, and the diapause pathway was chosen exclusively by a small subset of slow-growing individuals. Among the remainder, the choice of pathway was independent of the early growth rate; larvae entering diapause under the short daylength grew as fast as or faster than the direct developers under the longer daylength in the early instars, whereas the direct developers grew faster than the diapausers only in the ultimate instar. Hence, the pathway decision was state-dependent in a subset with a very high or very low growth rate, whereas the decision was state-independent in the majority of the larvae, which made the growth rate adjustment downstream from the pathway decision.

## Introduction

Generalist species often show different phenotypes in different environments due to either local genetic adaptations (Thompson 2005) or environmentally induced phenotypic plasticity (West-Eberhard 2003). Phenotypic plasticity can be either a passive result of the environmental conditions during development (e.g., sun exposure, temperature, food quality), or an adaptive response to the environment, with individuals making adaptive decisions based on information from the surrounding environment (e.g., Thompson 1991; Via 1993; Gotthard and Nylin 1995; Whitman et al. 2009). At the same time, the phenotypic response to different environments can be either a gradual reaction norm (e.g., Nijhout 2003; West-Eberhard 2003; Oostra et al. 2010) or the formation of two or several discrete phenotypes (e.g., Nijhout 2003; West-Eberhard 2003; Oostra et al. 2010) induced in different environments. The formation of these discrete phenotypes is typically the result of a pathway switch that is developmentally upstream from the future phenotype. After the developmental switch, which can be viewed as being equivalent to a decision point (Gotthard 2008), the different discrete phenotypes follow different developmental pathways. This results in a nonoverlapping variety of phenotypes in different environments (West-Eberhard 2003).

**Fig. 1:**
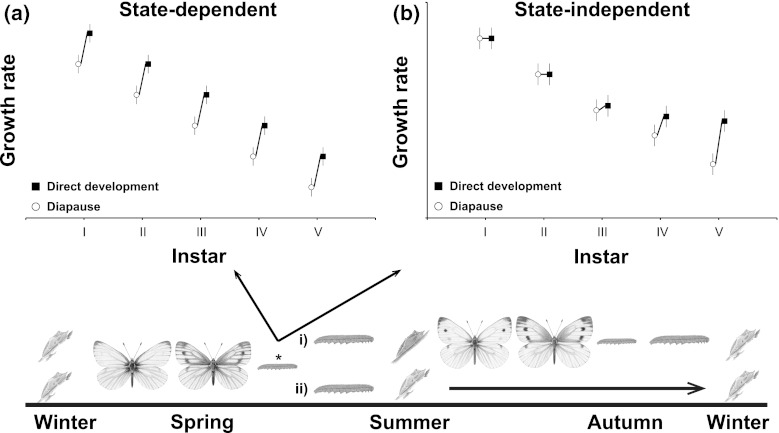
The lifecycle of the green-veined white butterfly in Sweden, and the predictions in the critical daylength experiment. The *asterisk* shows the offspring of the spring generation, which must decide in their fourth instar whether to enter (i) direct development and eclose as reproducing adults in the same season, or (ii) the diapause pathway and remain in the pupal stage until next spring. Direct developers typically show a higher overall growth rate than diapause developers, and the pathway decision is either (**a**) state-dependent if larvae that enter the direct development pathway (*filled squares*) grow faster than larvae entering diapause (*open circles*) throughout development (i.e., already before the pathway decision has been made) or (**b**) state-independent if direct developers (*filled squares*) grow faster than larvae set for diapause (*open circles*) only after the decision has been made in the fourth instar (Friberg et al. 2011). Butterfly illustrations by Richard Lewington (from Thomas and Lewington 2010)

In temperate areas, all organisms need a strategy to survive the cold winter months. Among insects, this period is typically spent in a species-specific diapause resting stage (Danilevskii 1965). The summer season is often of sufficient length to allow two or more reproductive generations, and in these systems, diapause is typically plastically induced as the onset of winter approaches. Species overwintering as eggs are often univoltine (e.g., Carrière et al. 1996; Saulich and Musolin 1996) or the diapause decision is maternally determined (e.g., Koevos and Tzanakakis 1991; Shintani and Higuchi 2008). In larval, pupal, and adult diapausers, the decision whether to enter diapause or to continue development to the reproductive stage without intermission is made during the larval period. The most reliable cue relating to seasonal progress is daylength, which is used by the majority of insects when choosing a developmental pathway (e.g., Danilevskii 1965; Yata et al. 1984; Eizaguirre et al. 1994). Another important cue is temperature, which is often less informative than daylength, as this cue varies quite drastically in a way that is less reliable in relation to seasonal progress (e.g., Danilevskii 1965; Eizaguirre et al. 1994; Friberg and Wiklund 2010), and in some phytophagous systems the pathway decision is also affected by the larval host plant (Hunter and McNeil 1997; Wedell et al. 1997; Friberg and Wiklund 2010). Long daylength, a high temperature, and high-quality host plants typically cue that it is favorable to continue development into yet another summer generation, whereas short daylength, a cold temperature, and poor-quality host plants indicate autumn conditions and foretell the onset of winter, meaning that it is more favorable to enter diapause. During a certain period at any given temperate latitude, the circumstances are such that half the broods of a bi- or multivoltine species make the decision to continue development, whereas the other half enters diapause, and when larvae are reared under such critical conditions it is commonplace that larvae that enter direct development show an overall higher growth rate than those entering diapause (Wiklund et al. 1991; Fig. 1). This pattern can be understood in an adaptive framework, as larvae under direct development are time stressed to reach adulthood and start reproduction so that their offspring will reach the overwintering stage before low temperatures make growth impossible, whereas the larval offspring of the spring generation under diapause development have ample time to reach the overwintering stage (Gotthard and Nylin 1995; Gotthard et al. 1999, 2000; Fig. 1).

The underlying ultimate factor that has selected for larval decision-making in temperate areas is the cold winter period, which makes certain physiological demands, such as withstanding below-zero temperatures (Hodkova and Hodek 2004). The overwintering diapause stage is phylogenetically conservative (Danilevskii 1965; Wiklund and Friberg 2011), which means that winter diapause for a given species can only be survived in a species-specific stage. Moreover, the preparations for winter diapause must be made well in advance of winter, so selection has favored the use of cues that make insects seemingly clairvoyant—they are able to foresee oncoming winter conditions. However, because an insect’s decision whether to develop directly or enter diapause development ultimately relates to the potential to reach the species-specific diapause stage before the onset of harsh conditions, cues like temperature and food quality can influence an individual’s prospective growth rate, and thereby yield relevant information on whether it is favorable to enter direct development. Whereas daylength informs the animal of the Julian date, and should be a reliable cue for seasonal position, temperature and host plant quality should be softer cues; nevertheless, both kinds of cues are bound to be important when an individual ectotherm decides whether it will be able to reach the diapause stage. Hence, pathway decision-making could integrate both state-independent and state-dependent aspects.

The difference in growth rates between larvae developing directly or under diapause development could thus have been implemented in two ways. If the decision is state-independent, the adjustment of growth rate occurs downstream of the developmental pathway decision, whereas the decision is state-dependent if larvae that grow slowly choose the diapause pathway and larvae that have a high growth rate choose the direct development pathway. In the study reported in this paper, our main objective was to clarify which of these alternatives apply by studying growth rate before and after the pathway decision in the green-veined white butterfly (*Pieris napi*).

### Predictions

*Pieris napi* overwinters as pupae (Fig. 1), and larvae develop through five larval instars before pupation. The period when larvae make their final developmental pathway decision in *P. napi* is located in the penultimate (IV) larval instar (Friberg et al. 2011). We made use of this information and inferred whether the growth rate adjustment for diapause or direct development is made downstream of the decision point (the state-independent hypothesis), or whether growth rate per se impacts on the decision (the state-dependent hypothesis). We did this using two experimental regimes: (1) by rearing larvae under two different daylengths, slightly shorter and slightly longer than the critical daylength, and calculating larval growth rates in each instar, and (2) by transferring larvae from a short (diapause-inducing) daylength to a long (direct development inducing) daylength and vice versa in the fourth instar, just when the pathway decision is made (Friberg et al. 2011). This way, we produced directly developing individuals that developed under both long and short daylength conditions.

In the first experiment (1), support for the state-dependent hypothesis would be provided if slow-growing larvae, particularly under the long daylength condition, were more likely to enter the diapause pathway, and if fast-growing larvae under the short daylength condition were more likely to enter the direct development pathway (Fig. 1a). Support for the state-independent hypothesis would be provided if larval growth rate was similar among all larvae before the decision point (Fig. 1b), and was only adjusted downstream of the decision point, so that directly developing larvae increased their growth rate in the later instars relative to larvae that chose the diapause development pathway (Fig. 1b). In the second experiment (2), support for the state-dependent hypothesis would be provided if larvae transferred to the short day environment could not maintain the same growth rate as larvae moved into the long day environment (a direct impact of daylength on growth rate), whereas the state-independent hypothesis would be supported if all directly developing individuals grew at similar rate, regardless of daylength condition.

## Materials and methods

### Study species

The green-veined white butterfly (*Pieris napi*) is a temperate butterfly present almost throughout the Northern Hemisphere (Eliasson et al. 2005) that is divided into different subspecies. It spends winter in pupal diapause, and in central Sweden it is generally bivoltine, but occasionally has a partial third generation in particularly warm summers (Eliasson et al. 2005). Eggs are laid singly on crucifers, and larvae develop through five larval instars before pupation.

In August 2008, 16 *P. napi* females were collected in the Stockholm area. Their offspring (>700 individuals) were reared to pupation in diapause-inducing conditions (16:8 h light/dark; 23°C) on a crucifer host plant (garlic mustard; *Alliaria petiolata*), and were incubated in a refrigerator maintained at −3°C until the following spring. Each experiment (see below) started with the transfer of a cohort of 60 pupae (ca 30 males and 30 females) to constant conditions (23°C). After terminating diapause, the adults eclosed and were released into a mating cage (0.8 × 0.8 × 0.5 m) with access to *Kalanchoe* sp. nectar plants sprayed with sugar solution. Most females mate during the first day of life in these cages (Larsdotter Mellström et al. 2010), and after two days *A. petiolata* host plants were presented to the females in the cage, which triggered egg-laying. Eggs were incubated on the host plants at room temperature until larval hatching, and the newly hatched larvae participating in each experiment were randomly chosen (weighing between 0.1 and 0.3 mg).

### Growth rate and pathway strategy under near-critical daylengths

The critical daylength for which about half of the *P. napi* larvae from the Stockholm area enter direct development while the other half enter diapause is typically around 18 h when larvae are reared at a temperature of 23°C (Wiklund et al. 1991; Wiklund and Friberg, unpublished data). The exact critical daylength is dependent on the temperature, and potentially also on light quality, and in order to avoid any unwanted effects of subtle differences between cabinets, both treatments in this experiment were performed in the same climate cabinet, using individuals randomly chosen from the same stock population (see above). In the first treatment, we applied a constant light regime of 18:6 h light/dark (23ºC) to 100 newly hatched *P. napi* larvae, and in the second treatment we applied an 18 h 15 min:5 h 45 min light regime (23°C) to another batch of 100 larvae. Larvae were reared individually in 0.5 L jars with ad libitum access to *A. petiolata*, and were weighed daily throughout development on a Cahn C-30 microbalance. The pupation date was noted, and pupae were weighed two days after pupation, when the pupal cuticle had hardened enough to allow handling. The direct developers were sexed at eclosion, whereas individuals that were classified as diapausing were sexed as pupae after having spent three weeks in 23°C without eclosing. The cabinet temperature was checked daily and was stable throughout the experiments.

### Transfer experiment

To further test for a growth rate adjustment downstream of the decision point, 100 newly hatched larvae were divided between two climate cabinets, each maintained at 23°C. One cabinet was set to induce diapause by applying a short daylength (16:8 h light/dark), whereas the other cabinet was set to induce direct development with a long daylength of 20:4 h light/dark (Friberg et al. 2011). All larvae were weighed daily, and as soon as a larva had reached the fourth instar it was transferred from its original daylength into the cabinet maintaining the alternative daylength. Hence, the larvae originally placed under the short day treatment were moved to the long day treatment as soon as they had reached the fourth instar, while the larvae originally placed under the long day treatment were moved to the short day treatment at the same developmental stage.

### Growth rate measurements and calculations

In all experiments, data on daily larval weights at the critical daylength were first transformed into individual growth rates. This was done by log-transforming all weight data, which allows the description of each larva’s growth trajectory as a linear function following the straight line equation (*y* = *kx* + *m*). The *k* value of the straight line can then be used as a measure of the average larval growth rate for each individual, and also for each individual’s growth rate in each of the five instars, by using the last weight of the previous instar as the start value and the first weight of the next instar as the end value. For example, the growth rate of a certain larva in the third instar was assessed by calculating the *k* value of the linear relationship including data from the last weight from instar two, all weights from instar three, and the first weight after the molt into instar four plotted against the number of days between the first and last data point. This allowed us to detect instar-specific growth rate differences between the two larval developmental pathways. The reason for including the weight measurements before and after each instar when calculating the instar-specific growth rate was that some larvae spent only one or a couple of days in one of the early instars. The fact that larval growth is not continuous but divided into five phases in accordance with the number of instars could also confound the results if the instar-specific growth rate were to be assessed with too few data points, as the growth rate slows down towards the end of each larval instar (cf. e.g., Nylin et al. 1989). This variation is likely to be especially prominent in the early larval stages and least important in the later instars, as these span a larger number of days. For the larvae of the transfer experiment, we calculated *k* (growth rate) of instars I–III (before the transfer) and instars IV–V (after the transfer).

### Statistical analysis

The data collected in the critical daylength experiment were tested in separate linear models with the average overall growth rate, the total development time (days from start of experiment to pupation), or the pupal weight as the response variable, and with the daylength (18 h/18 h 15 min), sex, and pathway (diapause/direct development) as categorical predictors. Nonsignificant interactions between factors were removed stepwise from the models.

Thereafter, the instar-specific development time (days/instar) and growth rate (log mg/day/instar) were tested in separate repeated-measures approaches (ANOVA III) using the GLM package in the statistical software Statistica 10 (StatSoft 2011). In each model, the growth rate or development time of each of the five instars was set as the repeatedly measured response variable, and was initially tested against the categorical predictor variables sex, pathway, daylength, and their interactions. Nonsignificant main effects and interactions were removed stepwise from the models. Individual instar-specific contrasts between pathways and daylengths were tested using Tukey’s honestly significant difference test to disentangle the roles of state-dependent vs. state-independent decision-making. In order to further visualize and test the relationship between the growth rate and the pathway decision in the different treatments, the individual growth rates were calculated (as described above) for each larva for the entire period before the decision was made (instar I–IV), and for the entire growth period remaining after the pathway decision (instar IV–V). Since a previous study has shown that the pathway decision is made during the fourth instar (Friberg et al. 2011), we included the daily weight data from instar IV in the calculations of growth rates both before and after the decision. These data were then tested in a logistic regression with growth rate before and after the pathway decision as the repeatedly measured response variable, with pathway and treatment as categorical predictor variables, and with logit as the link function. Nonsignificant interactions were removed stepwise from the final model.

The data obtained in the transfer experiment were analyzed in a repeated-measures ANOVA (III) with the growth rates of the direct developers from the two different treatments before (instars I–III) and after (IV–V) the transfer used as the repeatedly measured response variable, and with sex and transfer direction used as categorical predictors.

## Results

### Growth rate and pathway strategy under near-critical daylengths

The majority (68) of the 78 surviving larvae entered diapause when reared under an 18 h daylength; in the replicated experiment with a 15 min longer daylength, the result was largely reversed, with 79 larvae developing directly and 14 larvae entering diapause development (Table 1).

**Table 1:** Sample sizes (*n*), instar-specific mean development times and growth rates, and average pupal weights of different sexes and pathways (diapause/direct developers) under different daylengths (18 h and 18 h 15 min)

Daylength	Sex	Pathway	Instar	*n*	Development time (days)		Growth rate (log mg/day)		Pupal weight (mg)	
					Mean	Std dev	Mean	Std dev	Mean	Std dev
18 h	F	Diapause	I	38	2.86	±0.07	0.369	±0.006		
18 h	F	Diapause	II	38	1.45	±0.1	0.305	±0.006		
18 h	F	Diapause	III	38	2.08	±0.09	0.276	±0.006		
18 h	F	Diapause	IV	38	2.42	±0.1	0.240	±0.005		
18 h	F	Diapause	V	38	4.11	±0.1	0.186	±0.005		
18 h	F	Diapause	Total	38	12.92	±0.18	0.273	±0.004	153.6	±2.5
18 h	F	Direct	I	8	3.25	±0.15	0.368	±0.013		
18 h	F	Direct	II	8	1.13	±0.21	0.353	±0.014		
18 h	F	Direct	III	8	1.75	±0.19	0.292	±0.014		
18 h	F	Direct	IV	8	2.13	±0.21	0.261	±0.01		
18 h	F	Direct	V	8	3.63	±0.23	0.209	±0.01		
18 h	F	Direct	Total	8	11.88	±0.4	0.291	±0.009	168.5	±5.4
18 h	M	Diapause	I	30	2.83	±0.08	0.364	±0.007		
18 h	M	Diapause	II	30	1.63	±0.11	0.289	±0.007		
18 h	M	Diapause	III	30	2.07	±0.1	0.276	±0.007		
18 h	M	Diapause	IV	30	2.50	±0.11	0.239	±0.005		
18 h	M	Diapause	V	30	4.10	±0.12	0.185	±0.005		
18 h	M	Diapause	Total	30	13.13	±0.2	0.267	±0.005	160.3	±2.8
18 h	M	Direct	I	2	2.50	±0.31	0.371	±0.026		
18 h	M	Direct	II	2	1.50	±0.41	0.366	±0.028		
18 h	M	Direct	III	2	2.00	±0.38	0.306	±0.027		
18 h	M	Direct	IV	2	2.00	±0.42	0.265	±0.021		
18 h	M	Direct	V	2	3.00	±0.45	0.207	±0.021		
18 h	M	Direct	Total	2	11.00	±0.79	0.310	±0.018	169.2	±10.8
18 h 15 min	F	Diapause	I	4	2.50	±0.22	0.351	±0.018		
18 h 15 min	F	Diapause	II	4	2.00	±0.29	0.274	±0.019		
18 h 15 min	F	Diapause	III	4	2.00	±0.27	0.265	±0.019		
18 h 15 min	F	Diapause	IV	4	2.75	±0.3	0.225	±0.015		
18 h 15 min	F	Diapause	V	4	4.00	±0.32	0.184	±0.015		
18 h 15 min	F	Diapause	Total	4	13.25	±0.56	0.256	±0.013	143.1	±7.7
18 h 15 min	F	Direct	I	37	2.41	±0.07	0.334	±0.006		
18 h 15 min	F	Direct	II	37	2.24	±0.1	0.267	±0.006		
18 h 15 min	F	Direct	III	37	1.89	±0.09	0.286	±0.006		
18 h 15 min	F	Direct	IV	37	2.38	±0.1	0.243	±0.005		
18 h 15 min	F	Direct	V	37	3.03	±0.1	0.216	±0.005		
18 h 15 min	F	Direct	Total	37	11.95	±0.18	0.272	±0.004	156.3	±2.5
18 h 15 min	M	Diapause	I	10	2.10	±0.14	0.337	±0.012		
18 h 15 min	M	Diapause	II	10	2.50	±0.19	0.247	±0.012		
18 h 15 min	M	Diapause	III	10	2.00	±0.17	0.257	±0.012		
18 h 15 min	M	Diapause	IV	10	2.60	±0.19	0.224	±0.009		
18 h 15 min	M	Diapause	V	10	4.30	±0.2	0.194	±0.009		
18 h 15 min	M	Diapause	Total	10	13.50	±0.35	0.252	±0.008	162.2	±4.8
18 h 15 min	M	Direct	I	42	2.24	±0.07	0.346	±0.006		
18 h 15 min	M	Direct	II	42	2.21	±0.09	0.283	±0.006		
18 h 15 min	M	Direct	III	42	1.71	±0.08	0.299	±0.006		
18 h 15 min	M	Direct	IV	42	2.26	±0.09	0.267	±0.006		
18 h 15 min	M	Direct	V	42	2.86	±0.1	0.228	±0.004		
18 h 15 min	M	Direct	Total	42	11.29	±0.17	0.292	±0.004	168.5	±2.4

**Fig. 2:**
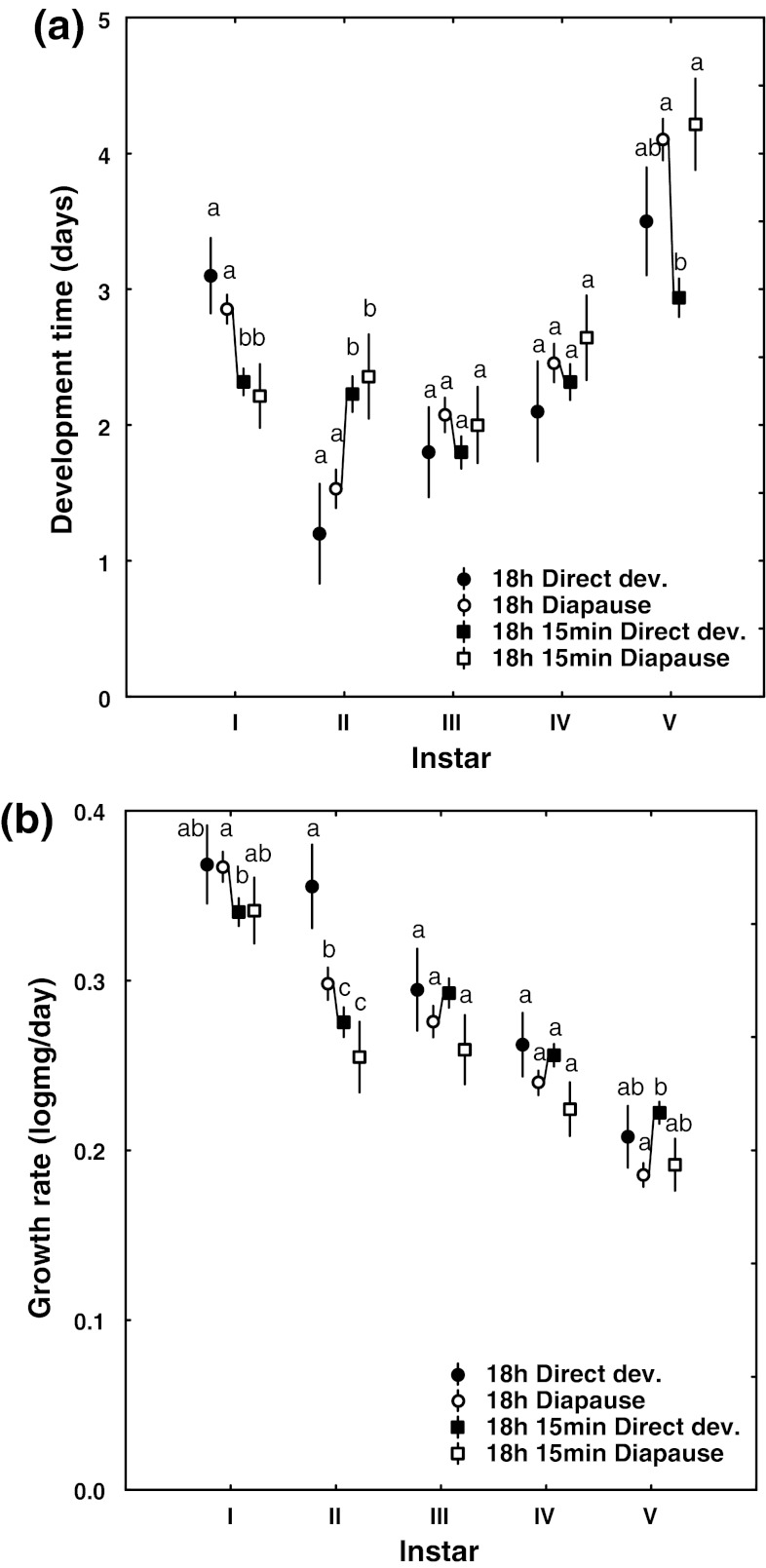
**a** Average number of days spent (±95% CI) in each larval instar and **b** the growth rate (log mg/day ± 95% CI) of each larval instar for directly developing (*filled circles*) and diapausing (*open circles*) individuals under an 18 h daylength, and for directly developing (*filled squares*) and diapausing individuals (*open squares*) under an 18 h 15 min daylength. The *line* highlights the differences in growth rate between the two focus groups of diapausing individuals under the 18 h daylength and directly developing individuals under the 18 h 15 min daylength. *Letters* above data points show post hoc significant patterns within each instar (groups with *different letters* are significantly different; Tukey’s HSD test)

The growth period from newly hatched larva to pupa varied between 10 and 17 days, with an overall average across treatments of 12.3 ± (SD) 1.35 days. Each larva spent roughly the first third of its larval life in instars I and II, the second third in instars III and IV, and the final third of the growth period in the last instar (V) (Table 1; Fig. 2a). The total development time was not significantly affected by the small difference in daylength (mean_18 h_ = 12.84 ± 1.22 days; mean_18 h 15 min_ = 11.87 ± 1.30 days; daylength *F*_1,167_ = 0.81, *P* = 0.37; Table 2), but larval development time was on average 1.5–2 days longer under diapause development versus direct development (mean_diapause_ = 13.09 ± 1.16 days; mean_direct dev_ = 11.61 ± 1.10 days; pathway *F*_1,167_ = 76.19, *P* < 0.001; Tables 1,2). Moreover, males developed faster than females, but only under direct development (for means see Table 1; sex × pathway *F*_1,167_ = 7.52, *P* = 0.0068; Table 2). Larval growth rate was higher under direct development (mean_diapause_ = 0.268 ± 0.023 log mg/day; mean_direct dev_ = 0.284 ± 0.029 log mg/day; pathway *F*_1,166_ = 27.09, *P* < 0.001), and especially males under direct development presented a higher growth rate than directly developing females and males and females under diapause development (for means, see Table 1; sex × pathway *F*_1,166_ = 10.82, *P* = 0.0012; Table 2). Moreover, the average growth rate differed between daylengths, as larvae grew slightly faster on average under the longer daylength treatment (mean_18 h_ = 0.273 ± 0.024 log mg/day; mean_18.15_ = 0.278 ± 0.030 log mg/day; daylength *F*_1,166_ = 9.38, *P* = 0.0026; Table 2). Pupal weight was higher under direct development (mean_diapause_ = 156.6 ± 18.6 mg; mean_direct dev._ = 163.4 ± 13.12 mg; pathway *F*_1,168_ = 8.37, *P* = 0.004), and male pupal weight was higher than female weight under both direct and diapause development (mean_males_ 164.9 ± 14.3 mg; mean_females_ = 155.6 ± 17.0 mg; sex *F*_1,168_ = 15.3, *P* < 0.001 Table 1, 2).

**Table 2:** ANOVA (III) table showing the effects of sex, larval pathway (diapause/direct development), daylength (18 h/18 h 15 min), and interactive effects between the different factors on the average pupal weight, development time (number of days between newly hatched larva and newly formed pupa), and growth rate (averaged over the entire larval period)

	Pupal weight				Development time				Growth rate			
	SS	*df*	*F*	*P*	SS	*df*	*F*	*P*	SS	*df*	*F*	*P*
Sex (S)	3629.4	1	15.3	**<0.001**	1.61	1	1.30	0.26	0.00191	1	3.04	0.083
Pathway (P)	1982.1	1	8.37	**0.004**	94.16	1	76.19	**<0.001**	0.01700	1	27.09	**<0.001**
Daylength (DL)	*425.0*	*1*	*1.81*	*0.18*	*1.020*	*1*	*0.812*	*0.37*	0.00589	1	9.38	**0.0026**
S × P	*149.3*	*1*	*0.64*	*0.43*	9.29	1	7.52	**0.0068**	0.00679	1	10.82	**0.0012**
S × DL	*522.4*	*1*	*2.22*	*0.14*	*0.059*	*1*	*0.047*	*0.83*	*0.000008*	*1*	*0.013*	*0.91*
P × DL	*17.41*	*1*	*0.074*	*0.79*	*0.106*	*1*	*0.084*	*0.77*	*0.000015*	*1*	*0.023*	*0.88*
S × P × DL	*0.55*	*1*	*0.023*	*0.96*	*0.029*	*1*	*0.023*	*0.88*	*0.000000*	*1*	*0.000*	*0.99*
Error	39782.2	168			206.4	167			0.10	166		

Data marked in italics denote main effects and interactions that were nonsignificant and removed from the final models

Significant *P* values are highlighted in bold font

At a general level, the effect of larval pathway on development time differed among the larval instars (for means, see Table 1; instar × pathway *F*_4,672_ = 11.0, *P* < 0.001, see Table 3 for full model; Fig. 2a), and the time spent in different instars also differed among the daylengths (instar × daylength *F*_4,672_ = 21.6, *P* < 0.001; Tables 1, 3; Fig. 2a). Within daylengths, there was no difference between direct developers and larvae set for diapause until the ultimate instar, which was about a day shorter for directly developing individuals than for the larvae set for diapause (Table 1; Fig. 2). The main comparison between the two larger groups (i.e., the diapausing individuals under the shorter daylength and the direct developers under the longer daylength) shows that development time was not consistently different until the ultimate instar; diapausing individuals spent on average more time in the first instar (Tukey’s HSD: *P* < 0.001; Fig. 2a) and a shorter time in the second instar than the direct developers (Tukey’s HSD: *P* < 0.001; Fig. 2a), whereas instars III and IV lasted a similar length of time for individuals set for diapause and direct development (Tukey’s HSD: *P*_III_ = 0.25; *P*_IV_ = 0.99; Fig. 2a). In the ultimate instar, individuals preparing for diapause spent about a day longer before pupating than the direct developers did (Tukey’s HSD: *P* < 0.001; Fig. 2a; Table 1).

**Table 3:** ANOVA (III) table showing the effects of larval instar (repeated measures), sex, daylength (18 h/18 h 15 min), larval pathway (diapause/direct development), and interactive effects between the different factors on the larval development time and growth rate

	Development time				Growth rate			
	SS	*df*	*F*	*P*	SS	*df*	*F*	*P*
Sex (S)	*0.21*	*1*	*0.84*	*0.36*	*0.00035*	*1*	*0.12*	*0.73*
Pathway (P)	10.7	1	41.6	**<0.001**	0.057	1	19.1	**<0.001**
Daylength (DL)	0.16	1	0.57	0.45	0.039	1	13.1	**<0.001**
S × P	*0.74*	*1*	*2.93*	*0.089*	*0.0052*	*1*	*1.78*	*0.18*
S × DL	*0.012*	*1*	*0.047*	*0.83*	*0.00017*	*1*	*0.058*	*0.81*
P × DL	*0.021*	*1*	*0.085*	*0.77*	0.000051	1	0.02	0.90
S × P × DL	*0.0058*	*1*	*0.023*	*0.88*	*0.00073*	*1*	*0.25*	*0.62*
Error	43.4	168			0.50	167		
Instar (I)	319.0	4	240.7	**<0.001**	1.05	4	331.6	**<0.001**
I × S	*2.78*	*4*	*2.09*	*0.080*	*0.0010*	*4*	*0.33*	*0.86*
I × P	14.5	4	11.0	**<0.001**	0.016	4	5.17	**<0.001**
I × DL	28.6	4	21.6	**<0.001**	0.058	4	18.4	**<0.001**
I × S × P	*0.71*	*4*	*0.54*	*0.71*	*0.0023*	*4*	*0.73*	*0.57*
I × S × DL	*0.78*	*4*	*0.59*	*0.67*	0.00095	4	0.30	0.88
I × P × DL	*0.79*	*4*	*0.59*	*0.67*	0.0086	4	2.74	**0.028**
I × S × P × SL	*1.55*	*4*	*1.17*	*0.32*	*0.00021*	*4*	*0.066*	*0.99*
Error	222.7	672			0.53	668		

Data marked in italics denote main effects and interactions that were nonsignificant and removed from the final models

Significant *P* values are highlighted in bold font

Across the entire sample, the instar-specific larval growth rate differed between daylengths and pathways (for means, see Table 1; instar × daylength *F*_4,668_ = 18.4, *P* < 0.001; instar × pathway *F*_4,668_ = 5.17, *P* < 0.001, Table 3; Fig. 2b), and there was a significant interaction indicating that the growth curves of the different pathways also differed between daylengths (instar × pathway × daylength *F*_4,668_ = 2.74, *P* = 0.028, Table 3; Fig. 2b), which is best illustrated by the direct developers under a daylength of 18 h, which showed a higher growth rate than the other groups from the early instars onwards (Fig. 2b).

As described above, it is important to compare the more common responses under each daylength (diapausers under the 18 h daylength, direct developers under the 18 h 15 min daylength) in order to determine the presence of state-independent strategic pathway decision-making. Such a comparison shows that the growth rate differences between pathways varied in intensity and direction in different instars. In the early instars, individuals that later chose the diapause development pathway grew even faster than those that later chose the direct development pathway in the early instars (Tukey’s HSD test *P*_I_ < 0.001; *P*_II_ = 0.014) and as fast as the direct developers in instars III and IV (Tukey’s HSD test *P*_III_ = 0.28; *P*_IV_ = 0.35), and the directly developing individuals grew significantly faster than those set for diapause only in the ultimate instar (Tukey’s HSD test *P*_V_ < 0.001; Fig. 2b).

**Fig. 3:**
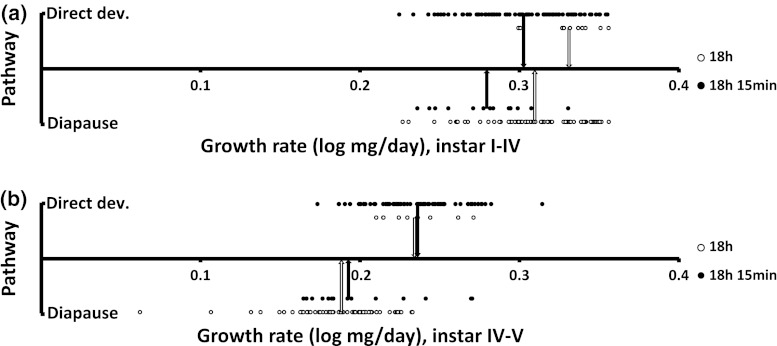
The individual growth rates of diapausing and directly developing individuals of larvae reared under a daylength of 18 h (*open circles*) or 18 h 15 min (*filled circles*: **a** Instars I–IV (before the pathway decision was made); **b** instars IV and V (after the pathway decision had been made). Each *arrow* shows the average growth rate for a group of larvae

Growth rate, measured as a repeated measure variable before and after the pathway decision point, was dependent on larval pathway and daylength treatment (rep. meas. ANOVA: daylength (DL) *F*_1,168_ = 5.69, *P* = 0.018; pathway (P) *F*_1,168_ = 38.7, *P* < 0.001; growth period (GP) *F*_1,168_ = 1707.0, *P* < 0.001; DL × GP *F*_1,168_ = 25.18, *P* < 0.001; P × GP* F*_1,168_ = 11.5, *P* < 0.001; additional interactions were nonsignificant—all *P* values >0.77—and were removed stepwise from the model). In more detail, larval growth rates before the pathway decision was made (in instars I–IV) were slowest among the larvae that entered diapause under the long day treatment, and highest among the larvae that entered direct development under the short day treatment (Tukey’s HSD: *P* < 0.001; Fig. 3), whereas there was no significant difference in growth rates between those that later entered diapause under the short daylength treatment and those that later entered direct development under the longer daylength treatment (Tukey’s HSD: *P* = 0.86; Fig. 3). This contrasts with the growth rate pattern after the decision had been made (instars IV–V), when there was no difference in diapausing individuals between treatments (Tukey’s HSD: *P* = 0.99) nor in the larvae set for direct development between the daylength treatments (Tukey’s HSD: *P* = 0.99), whereas growth rates differed significantly between larvae of the two different pathways within each daylength treatment (Tukey’s HSD: *P*_18 h_ < 0.001; *P*_18 h 15 min_ < 0.001; Fig. 3).

### Transfer experiment

**Fig. 4:**
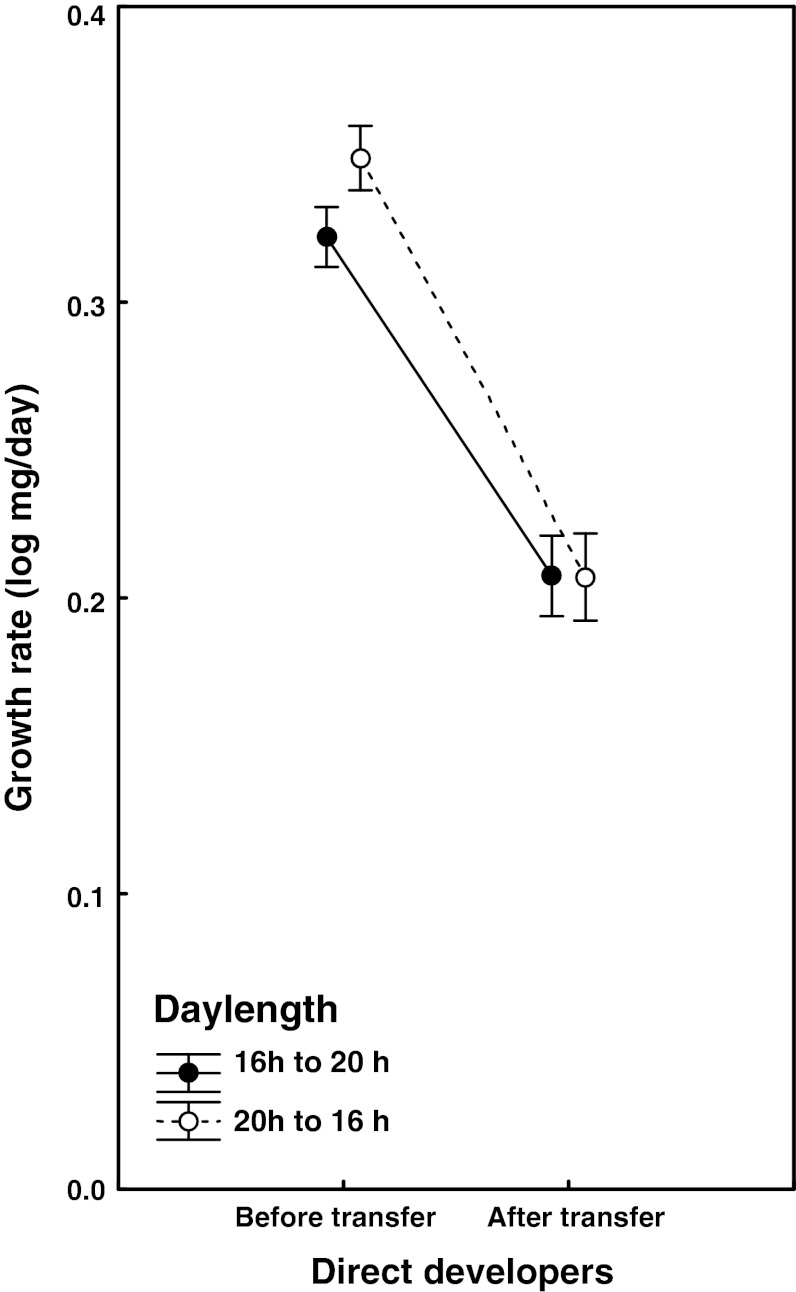
The larval growth rates (log mg/day ± 95% CI) of directly developing individuals before and after a transfer in the fourth instar from the long to the short daylength treatment (*open circles*) or in the opposite direction (*filled circles*)

Thirty-seven of the 42 surviving individuals that were transferred from the long to the short day environment developed directly, whereas 43 of the 45 larvae that were transferred in the opposite direction followed the same pathway. These directly developing individuals were used in the forthcoming analysis. Larval growth rate decreased with larval development stage (mean_instar I–III_ = 0.335 ± 0.036 log mg/day; mean_instar IV–V_ = 0.207 ± 0.028 log mg/day; growth period: *F*_1,77_ = 1557.9, *P* < 0.001; for full model, see Table 4; Fig. 4), reflecting how difficult it is in general for an insect larva to maintain the same proportional growth rate throughout development. More interestingly, before the transfer, larvae growing under the long day treatment in instars I–III showed a significantly higher growth rate than larvae initially reared under the short day treatment (mean_long DL_ = 0.349 ± 0.027 log mg/day; mean_short DL_ = 0.322 ± 0.038 log mg/day), whereas there was no difference in growth rate between the two daylength treatments in instars IV–V after the transfer (mean_long DL_ = 0.207 ± 0.024 log mg/day; mean_short DL_ = 0.207 ± 0.030 log mg/day; growth period × transfer direction: *F*_1,77_ = 18.9, *P* < 0.001, Table 4). Hence, directly developing larvae grew equally fast under both 16 and 20 h daylengths in the fourth and fifth larval instars, after the pathway decision had been finalized (Fig. 4). Females grew as fast as males in instars I–III (mean_males_ = 0.335 ± 0.037 log mg/day; mean_females_ = 0.334 ± 0.035 log mg/day), whereas males grew faster than females in instars IV–V (mean_males_ = 0.217 ± 0.024 log mg/day; mean_females_ = 0.200 ± 0.027 log mg/day; growth period × sex: *F*_1,77_ = 7.12, *P* = 0.0093; Table 4).

**Table 4:** ANOVA (III) table showing the effects of growth period (instars I–III or instars IV–V), sex, transfer direction, and interactive effects between the different factors on larval growth rates of directly developing larvae transferred from either the long to the short daylength treatment or vice versa in the fourth larval instar

	Growth rate			
	SS	*df*	*F*	*P*
Sex (S)	0.0024	1	1.72	0.19
Transfer direction (TD)	0.0065	1	4.67	**0.034**
S × TD	0.00003	1	0.022	0.88
Error	0.11	77		
Growth period (GP)	0.63	1	1557.9	**<0.001**
GP × S	0.0029	1	7.12	**0.0093**
GP × TD	0.0077	1	18.9	**<0.001**
GP × S × TD	*0.00044*	*1*	*1.08*	*0.30*
Error	0.031	77		

Data marked in italics denote main effects and interactions that were nonsignificant and removed from the final models

Significant *P* values are highlighted in bold font

## Discussion

The major incentive for this study was to determine the causal factor for the higher larval growth rate under direct development compared to diapause development. In particular, we have investigated whether this growth rate adjustment reflects a state-independent pathway choice and is implemented downstream of the developmental pathway decision point, or whether the decision is in itself state-dependent, so that larvae that grow slowly choose the diapause pathway while larvae that grow rapidly choose direct development.

### State-independent decision-making

The results from our experiments allow us to conclude that both processes appear to apply. The response to the slight increase in daylength (from 18 h to 18 h 15 min) was dramatic, with the lion’s share of the larvae reared under the shorter daylength entering diapause and the vast majority of the larvae reared under the longer daylength entering direct development. This strong response to the small daylength difference, and the observation that these groups show similar overall growth rates before the pathway decision is made in instar IV but significantly different growth rates after the decision has been made, strongly imply a state-independent background to the pathway decision (Figs. 1, 2, 3). The larvae thus make their pathway decision and then alter the growth rate in accordance with that decision. When comparing the groups instar by instar, the growth rate of the larvae set for direct development was significantly lower than the growth rate of the larvae set for diapause in the first two instars, whereas there were no significant differences in the growth rates in the third and fourth instars (when the pathway decision is made; Friberg et al. 2011). In the ultimate instar, larvae set for direct development grew significantly faster than those set for diapause (Fig. 2b), as predicted by the state-independent hypothesis (Fig. 1b). We can thus conclude that the larval pathway decision among these larvae is a case of state-independent decision-making, and that these larvae altered their growth rates as a response to their choice of pathway. The lower growth rates during early instars of larvae that later entered direct development (Fig. 2b) were not predicted, but are potentially explained by small inconsistencies in the timing of the daily weighing schedules between treatments. This interpretation is supported by the large variation in the durations of larval instars I and II in the different treatments (Fig. 2a) and the fact that the results of the follow-up analysis of the average growth rate across instars I–IV did not differ between these groups, whereas the post-decision growth rates of larvae set for direct development in the 18 h 15 min daylength treatment vastly exceeded the growth rates of larvae set for diapause in the 18 h daylength treatment (Fig. 3).

### State-dependent decision-making

Within each daylength treatment, there was a small group making the opposite decision than the majority, that is developing directly in 18 h daylength or entering diapause in 18 h 15 min. The larvae that entered direct development under the shorter daylength were the fastest growers in instar I–IV (before the critical stage for the pathway decision; Friberg et al. 2011), whereas the larvae that entered diapause under the longer daylength treatment had the slowest average growth rate during the same period. This result implies that the pathway decision is likely also guided to a certain extent by state-dependent decision-making. The decision to enter direct development might thus not be open to the slowest-growing larvae under a daylength slightly longer that the critical daylength, while the same pathway alternative is open for the fastest-growing individuals, even under a daylength slightly shorter than the critical daylength when half of the population enters each pathway. Previous support for state-dependent seasonal pathway decision-making comes from studies showing the importance of larval host plant quality or suitability in the pathway decision (Wedell et al. 1997; Hunter and McNeil 1997; Goehring and Oberhauser 2002), with larvae observed to more easily enter direct development on nutritionally superior host plants, which suggests that direct development is an option only when the host plant is nutritious enough to support a high growth rate.

Also in the transfer experiment, it is obvious that daylength had a more direct effect on the growth rate of the larvae, but only before the pathway decision was made. Larvae reared under a 16 h daylength in instars I–III grew significantly slower than larvae reared under a 20 h daylength. Interestingly, this effect disappeared after the pathway decision had been made in the fourth instar, and the larvae set for direct development showed similar growth rates under both 16 h and 20 h daylengths (Fig. 4). Alternatively, the slower growth rate in the early instars reflects a conditional early decision to enter diapause under the 16 h treatment that was reversed when these larvae were transferred to the long day treatment. It is important to remember that the decision point reported in a previous study (Friberg et al. 2011) must be interpreted as a point of no return that must have been preceded by a period of light period sensibility that lasted for at least 24 h (cf. Nijhout 2003). This period could, however, have lasted longer, and future studies are needed to investigate whether larvae actually make a conditional, tentative decision as small larvae, adjust their growth rates to fit that decision, but also possibly adjust that decision to fit the circumstances when entering the penultimate instar. In such a scenario, it is possible that larvae under the 16 h treatment made an early decision to aim for the diapause pathway, but had the opportunity to change that decision when transferred to the long daylength treatment as fourth-instar larvae.

### Growth rate increase/decrease under direct/diapause development

Although this study has disentangled cause from effect when it comes to the differences in development rate between diapause and direct developers, it is still largely unclear whether diapause developers decrease their growth rates, or direct developers actively increase theirs. Either scenario is easily envisaged from an adaptive perspective. Under the critical daylength, selection for early eclosion into adulthood is quite likely, since the sooner the adults eclose, the more time they have to reproduce and sire offspring that, in turn, need enough time to reach the critical pupal stage before the onset of winter. This scenario seems to apply to directly developing males in our experiment which had a significantly higher growth rate than directly developing females and males and females under diapause development. This sex-specific increased growth rate in male *P. napi* has been reported previously (Wiklund and Forsberg 1991), and it was argued that this is an adaptive response to the dual selective pressures on males to emerge before females (i.e., selection for protandry; Wiklund and Fagerström 1977) and to grow large so that they can transfer a large nuptial gift to the female during mating (Wiklund and Kaitala 1995). On the other hand, the environmental demands on diapausing pupae are severe, and it is likely that larvae set for diapause simply cannot grow as quickly as direct developers, so they must decrease their growth rate in order to prepare themselves for the long and cold winter months. The latter interpretation is supported by the observation that it appears to be more difficult to switch from being set for direct development to entering diapause than vice versa (Friberg et al. 2011), and we contend that the higher developmental demands facing individuals that are set for diapause are likely to be of general importance for bi- and multivoltine insects in temperate areas (see e.g., Friberg and Karlsson 2010).

Hence, once again it appears that an either/or scenario is inappropriate, just as decision-making when it comes to the question of whether to diapause or not includes both state-independent and state-dependent decisions. It seems likely that both alternatives apply when it comes to growth rates under diapause or direct development, so that growth rate is increased under direct development and conversely decreased under diapause development towards the end of the development period, when diapause-specific physiological adaptations may have to be implemented.

### The pathway decision as a developmental switch

Many insects, including *P. napi* (e.g., Karlsson and Johansson 2008; Larsdotter Mellström et al. 2010), have evolved seasonal polyphenism, which means that different seasonal cohorts or generations show different discrete phenotypes (Shapiro 1976; Nijhout 2003; West-Eberhard 2003). These phenotypes could be positioned at different locations of a continuous reaction norm to environmental variation (Nijhout 2003; Oostra et al. 2010), but they could also be examples of canalized discrete phenotypes (Nijhout 2003; Oostra et al. 2010), where the developmental pathways of several phenotypic traits are induced at the same time. This decision point is termed a “developmental switch” (Nijhout 2003; West-Eberhard 2003; Gotthard 2008). Whereas several studies have investigated the consequences and the environmental cues and hormone control mechanisms of importance for the developmental switch (Nijhout 2003; West-Eberhard 2003; Gotthard 2008), fewer studies have linked the mechanisms to the life-history evolution underlying the actual developmental switch itself. One of the best examples of a group of studies that make the full connection from cue to mechanism to life history is Emlen and Nijhout’s work, which showed the entire process leading to different discrete phenotypes (males with and without horns) in the dung beetle *Onthophagous taurus* (Emlen 1997; Emlen and Nijhout 1999, 2001; Nijhout 2003). They determined the hormonal control mechanism and the environmental cues responsible for the state-dependent developmental switch (Emlen and Nijhout 1999, 2001), as well as the life-history evolution that ultimately selects for the alternative phenotypes (Emlen 1997). By increasing our knowledge of the mechanisms that control the developmental switch and the ultimate selection pressures that generate the canalized phenotypes, it will be possible to generate hypotheses about how future selection will affect the switching mechanism and the speed with which phenotypically plastic organisms can adapt to changing environments.

The data reported in this study have shed further light on the actual pathway decision-making process and its links to life-history biology and evolutionary adaptation. Most importantly, this study shows how an external, state-independent cue (light period sensitivity) interacts with an internal, state-dependent cue (larval growth rate) to shape the larval pathway decision and the induction of seasonal phenotypes. Our increased understanding of the dynamics of the developmental and ecological processes that affect the developmental switch in *P. napi* butterflies also generates new questions about the abundance and distribution of genetic variation in pathway decision-making, and the potential for selection on the actual developmental switch to quite rapidly affect the phenotypic evolution of this and other polyphenic species.
